# Computational Prediction of Antiangiogenesis Synergistic Mechanisms of Total Saponins of *Panax japonicus* Against Rheumatoid Arthritis

**DOI:** 10.3389/fphar.2020.566129

**Published:** 2020-10-29

**Authors:** Xiang Guo, Jinyu Ji, Goutham Sanker Jose Kumar Sreena, Xiaoqiang Hou, Yanan Luo, Xianyun Fu, Zhigang Mei, Zhitao Feng

**Affiliations:** ^1^ Third-Grade Pharmacological Laboratory on Chinese Medicine Approved by State Administration of Traditional Chinese Medicine, Medical College of China Three Gorges University, Yichang, China; ^2^ Institute of Rheumatology, the First College of Clinical Medical Sciences, China Three Gorges University, Yichang, China; ^3^ Key Laboratory of Hunan Province for Integrated Traditional Chinese and Western Medicine on Prevention and Treatment of Cardio-Cerebral Diseases, Hunan University of Chinese Medicine, Changsha, China

**Keywords:** *Panax japonicus*, saponins, rheumatoid arthritis, angiogenesis, network pharmacology

## Abstract

**Objective:** To investigate the anti-angiogenesis mechanisms and key targets of total saponins of *Panax japonicus* (TSPJ) in the treatment of rheumatoid arthritis (RA).

**Methods:** RStudio3.6.1 software was used to obtain differentially expressed genes (DEGs) by analyzing the differences in gene expression in the synovial tissue of RA and to predict the potential targets of active compounds from TSPJ by the PharmMapper and SwissTargetPrediction databases. We evaluated the overlapping genes by intersectional analysis of DEGs and drug targets. Based on the overlapping genes, we used Cytoscape 3.7.2 software to construct a protein–protein interactions (PPI) network and applied Kyoto Encyclopedia of Genes and Genomes (KEGG) analysis to determine the mechanisms of the treatment. Finally, the correlations with angiogenesis-related genes were explored. Collagen-induced arthritis (CIA) model was established and treated with different doses of TSPJ. The manifestations of CIA were determined by evaluation of arthritis index and histology score. Serum levels of vascular endothelial growth factor (VEGF) and the hypoxia-inducible factor 1 (HIF-1) were tested by ELISA. The mRNA levels of IL-1β and IL-17A were detected by real time-quantitative PCR.

**Results**: Altogether, 2670 DEGs were obtained by differential analysis, and 371 drug targets were predicted for four active components (Araloside A, Chikusetsusaponin IVa, Ginsenoside Rg2, and Ginsenoside Ro). A total of 52 overlapping genes were included in the PPI network and the KEGG analysis. However, only 41 genes in the PPI network had protein interactions. The results of the KEGG enrichment analysis were all related to angiogenesis, including VEGF and HIF-1 signaling pathways. Seven genes with negative correlations and 16 genes with positive correlations were obtained by correlational analysis of DEGs in the VEGF and HIF-1 signaling pathways. SRC proto-oncogene, nonreceptor tyrosine kinase (SRC), and the signal transducer and activator of transcription 3 (STAT 3) had a higher value of degree and showed a significant correlation in the pathways; they were regarded as key targets. Compared with the model group, TSPJ significantly relieved the symptoms and decreased the expression of VEGFA, HIF-1α, IL-1β, and IL-17A in serum or spleens of CIA mice.

**Conclusion:** In the current study, we found that antiangiogenesis is one of the effective strategies of TSPJ against RA; SRC and STAT 3 may be the key targets of TSPJ acting on the VEGF and HIF-1 signaling pathways, which will provide new insight into the treatment of RA by inhibiting inflammation and angiogenesis.

## Introduction

Rheumatoid arthritis (RA) is a chronic inflammatory autoimmune disease affected by both genetic and environmental factors (Croia et al., 2019). Approximately 0.5–1% of the world’s population suffers from RA, and many patients have symptoms of joint damage or even disability (Smolen et al., 2016; Aletaha and Smolen, 2018). When synovitis occurs, macrophages and fibroblasts in synovial tissue are induced to secrete angiogenic factors, leading to an abnormal increase of synovial vessels. A large number of inflammatory cells enter the joint through newly formed blood vessels, causing synovitis and synovial hyperplasia. Increased angiogenesis leads to the permanence of RA (Elshabrawy et al., 2015; Leblond et al., 2017; Lu et al., 2017; Chen et al., 2018). Eventually, these mechanisms destroy the joint and lead to deformity. It is worth noting that the treatment of RA with vascular targeted therapy has gradually entered the clinic (Leblond et al., 2017).

Recently, many people have become interested in research into traditional Chinese medicine (TCM) because it plays an important role in the treatment of difficult and complicated diseases (Yuan et al., 2017). The study of Chinese herbal medicine, with the identification of the main components of herbals and the molecular mechanisms of their curative effects, has become a hot research topic. Currently, TCM is increasingly being used in clinical treatment or for developing new treatment methods (Wang et al., 2018). *Panax japonicus* (T.Nees) C.A.Mey is a kind of Chinese herbal medicine that belongs to the *Panax* genus, which is widely grown in the southwest of China. Studies have shown that *Panax japonicus* (T.Nees) C.A.Mey has analgesic, anti-inflammatory, antioxidant, and joint swelling-relieving properties (Yang et al., 2014; Yang et al., 2018). Saponins are the main components of *Panax japonicus* (T.Nees) C.A.Mey. Total saponins of *Panax japonicus* (TSPJ) are extracted from *Panax japonicus* (T.Nees) C.A.Mey and are widely used to treat RA. Its main ingredients include Araloside A, Chikusetsusaponin IVa, Ginsenoside Rg2, and Ginsenoside Ro (Yu-min et al., 2019) (Supplementary Figure S1 and Table S1). Ding et al. have confirmed that Araloside A has an anti-inflammatory effect in the treatment of RA (Ding et al., 2019). Chikusetsusaponin IVa and Ginsenoside Ro exhibit antiangiogenic effects (Zheng et al., 2019). In the treatment of RA, TSPJ can inhibit synovial hyperplasia and reduce capillary permeability (Guo et al., 2019). Yuan et al. found that TSPJ could reduce the degree of foot swelling (Ding et al., 2008) and downregulate the serum levels of TNF-α and IL-1β in CIA rats (Ding et al., 2009). Related studies have shown that TSPJ can inhibit angiogenesis during tumor therapy (Yougui, 2011). However, no previous studies have shown whether TSPJ can be used as a targeted drug against angiogenesis in RA.

**FIGURE 1:**
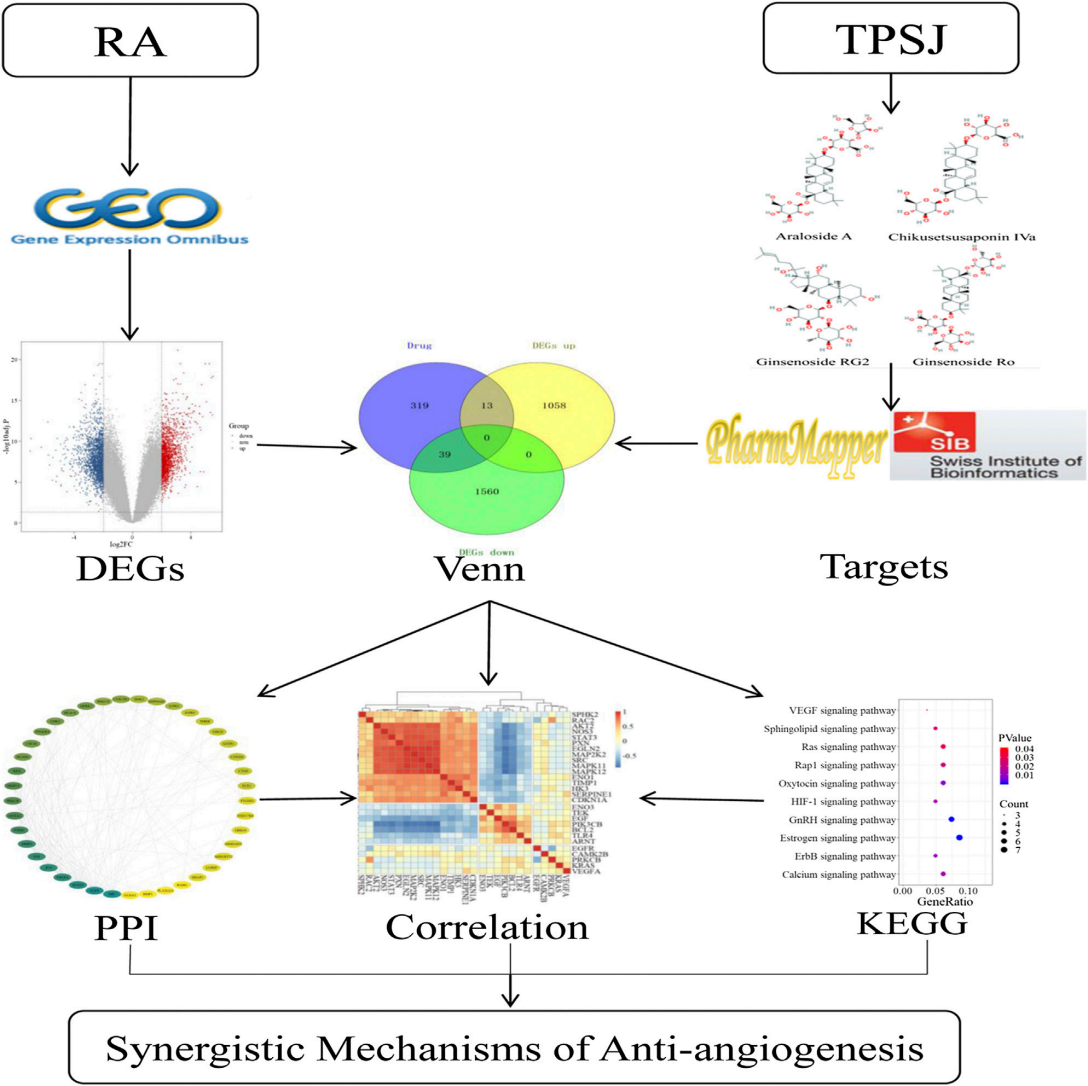
General research framework. DEGs, differentially expressed genes; KEGG, Kyoto Encyclopedia of Genes and Genomes; PPI, protein–protein interactions network; RA, rheumatoid arthritis; TSPJ, total saponins of *Panax japonicus*.

With the development of biomedical big data research, the network pharmacology approach and data mining in bioinformatics is being applied to help study the potential mechanism of different actions of drugs in organisms (Boezio et al., 2017) and has been proven to effectively reveal the regulation principles of small molecules of TCM (Zhang R.-z. et al., 2017). There is a large quantity of biological data in the public database, among which there is much valuable information that has not yet been highlighted. Bioinformatic methods are widely used to extract productive data because of their advantages of being quick and inexpensive (Ramharack and Soliman, 2018). By constructing an interaction network of disease-related proteins of TCM components, network pharmacology can identify the key targets in the network, and study the interactions among the drugs, targets, and pathways (Yuan et al., 2017; Sidders et al., 2018). In this study, we first identified differentially expressed genes (DEGs) of RA related to angiogenesis by bioinformatics analysis. After predicting potential targets for the active compounds of TSPJ, we used a network pharmacology approach to identify the possible antiangiogenic mechanism of the main components of TSPJ in RA therapy. Because CIA model has many similar pathological and immunological characteristics to human RA, many animal experiments have used CIA mice as RA models (Choudhary et al., 2018). We successfully established CIA model and verified partly of our computational prediction results (Supplementary Figures S2–S5). The findings of this study deepen our understanding of the therapeutic mechanism of TSPJ and provide a new therapeutic idea in the antiangiogenic treatment of RA. The flow diagram of this study is shown in Figure 1.

## Methods

### Data Collection and Preprocessing

The Gene Expression Omnibus (GEO) database (https://www.ncbi.nlm.nih.gov/geo/), a public functional genomics database built and maintained by the National Center for Biotechnology Information (NCBI), collects microarray, next-generation sequencing, and other forms of high-throughput functional genomic data. Persistent synovitis and joint destruction are the main characteristics of RA (Scott et al., 2010). In our study, we mainly studied the difference in synovial tissue expression between RA patients and healthy individuals. Microarray raw data were downloaded from the GEO database by searching with the keywords: “rheumatoid arthritis,” “synovial tissue,” and “*Homo sapiens*.” All the above data sets were merged according to the same probe name by RStudio3.6.1 software to build the gene expression data matrix. The gene expression data matrix was normalized using the “Limma” R package. In the end, the gene expression matrix of synovial tissue was obtained.

PubChem (https://pubchem.ncbi.nlm.nih.gov/) is an open-access database that contains chemical structures, molecular formulas and patents, and article identifiers of drugs (Sayers et al., 2020). The chemical structures of the main components of TSPJ were searched in the PubChem database. Based on the chemical structure models, drug targets were predicted via the PharmMapper database (http://www.lilab-ecust.cn/pharmmapper/) and the SwissTargetPrediction database (http://www.swisstargetprediction.ch/).

### Differentially Expressed Genes' Screening

The “Limma” R package is commonly used for variance analysis. First, RStudio3.6.1 software was used to read the gene expression matrix of synovial tissue. All samples were divided into two groups (healthy control and the RA group). Second, the “Limma” package was adopted to identify the DEGs between the healthy control and the RA group. The results also included fold change (FC) and the *p* values to reflect the degree of gene differences. *p* values were adjusted for multiple test correction using the Hochberg and Benjamini test. Finally, |log2FC| > 2 and adj.*p* < 0.05 were used as the cutoff criteria to identify DEGs.

### Network Pharmacology

The UniProt database (http://www.uniprot.org/) provides high-quality annotation for proteins (The UniProt Consortium, 2017). The UniProt database was utilized for retrieving drug prediction targets information and screening out targets for the “*Homo sapiens*” species. The overlapping targets between the drug targets and the DEGs were identified, and input into the STRING database (https://string-db.org/) to retrieve the protein–protein interactions (PPI). Cytoscape3.7.2 software was used to import the data to construct and visualize the PPI network. We used the “Network Analyzer” plugin to analyze the network to obtain network-related parameters.

### Pathway Enrichment Analysis

The DAVID database (https://david.ncifcrf.gov/) is commonly used for annotation, visualization, and integrated discovery (Dennis et al., 2003). To illustrate the possible mechanism of drug action in the treatment of RA, the overlapping targets were adopted for the Kyoto Encyclopedia of Genes and Genomes (KEGG) enrichment analysis by the DAVID database with the “*Homo sapiens*” setting. Finally, the signaling pathway with *p* < 0.05 was selected for the next study.

### Correlation Analysis

Significant signaling pathways were acquired by KEGG enrichment analysis. To learn more about the synergistic mechanisms of these pathways, we performed correlation analysis and identified negative and positive genes in the related pathways. First, we downloaded all targets in these signaling pathways from the KEGG (https://www.kegg.jp/) database. Second, the overlapping genes between the DEGs and pathways were identified by intersection analysis. Finally, the roles of these overlapping genes in the pathway were determined by correlation analysis.

## Results

### Gene Expression Matrix

After a systematic review, we found two gene expression data sets that met our requirements. The gene expression data sets GSE48780 and GSE77298 were downloaded from the GEO database (Supplementary Table S2). These data sets are based on the GPL570 platform (Affymetrix Human Genome U133 Plus 2.0 Array). We used the “dplyr” R package to merge the data sets to construct the gene expression matrix of synovial tissue. This matrix contained seven healthy samples and 99 RA samples.

### Differentially Expressed Genes

**FIGURE 2:**
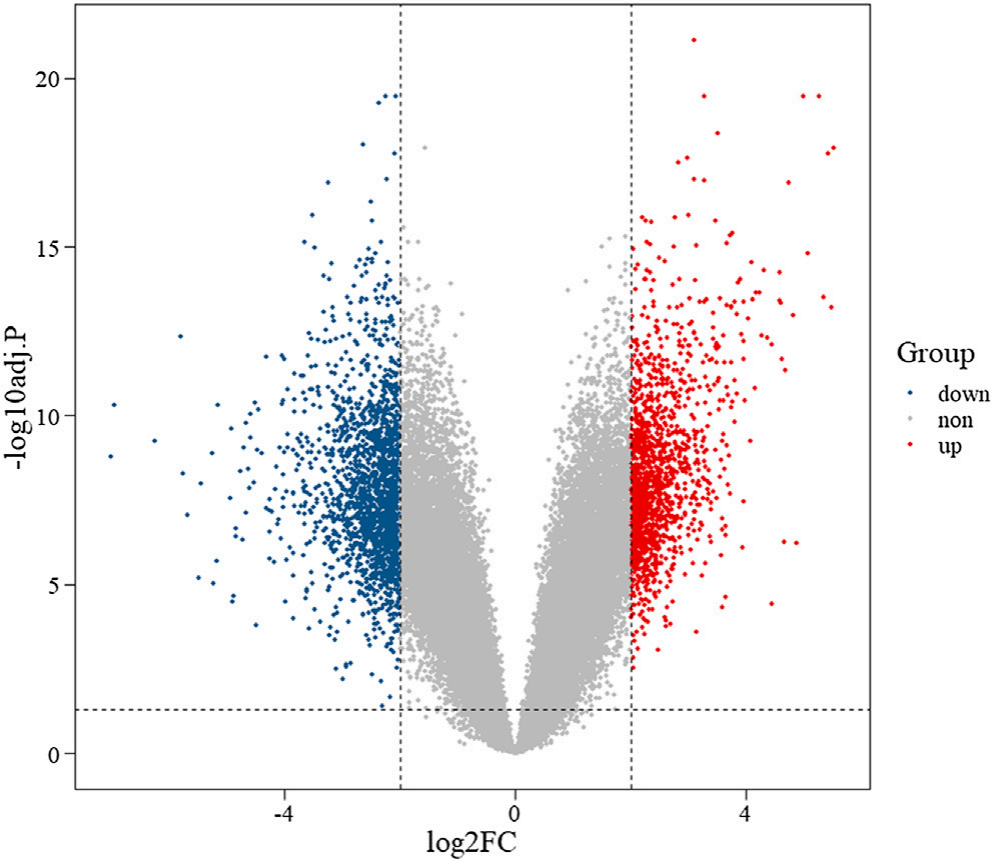
Volcano plot of differentially expressed genes. Note: red represents upregulated genes, blue represents downregulated genes, and gray represents background genes. *X*-axis represents log fold change, and *Y*-axis represents log 10-adjusted *p*-value.

We compared two groups of data in the gene expression matrix, and subsequently we obtained 2,670 DEGs (adj.*p* < 0.05, |log2FC| > 2), including 1,071 upregulated (adj.*p* < 0.05, log2FC > 2) and 1,599 downregulated (adj.*p* < 0.05, log2FC < −2) DEGs. Meanwhile, the volcano plot was used to display both the DEGs and the background genes (Figure 2).

### Identification of the Overlapping Targets

**FIGURE 3:**
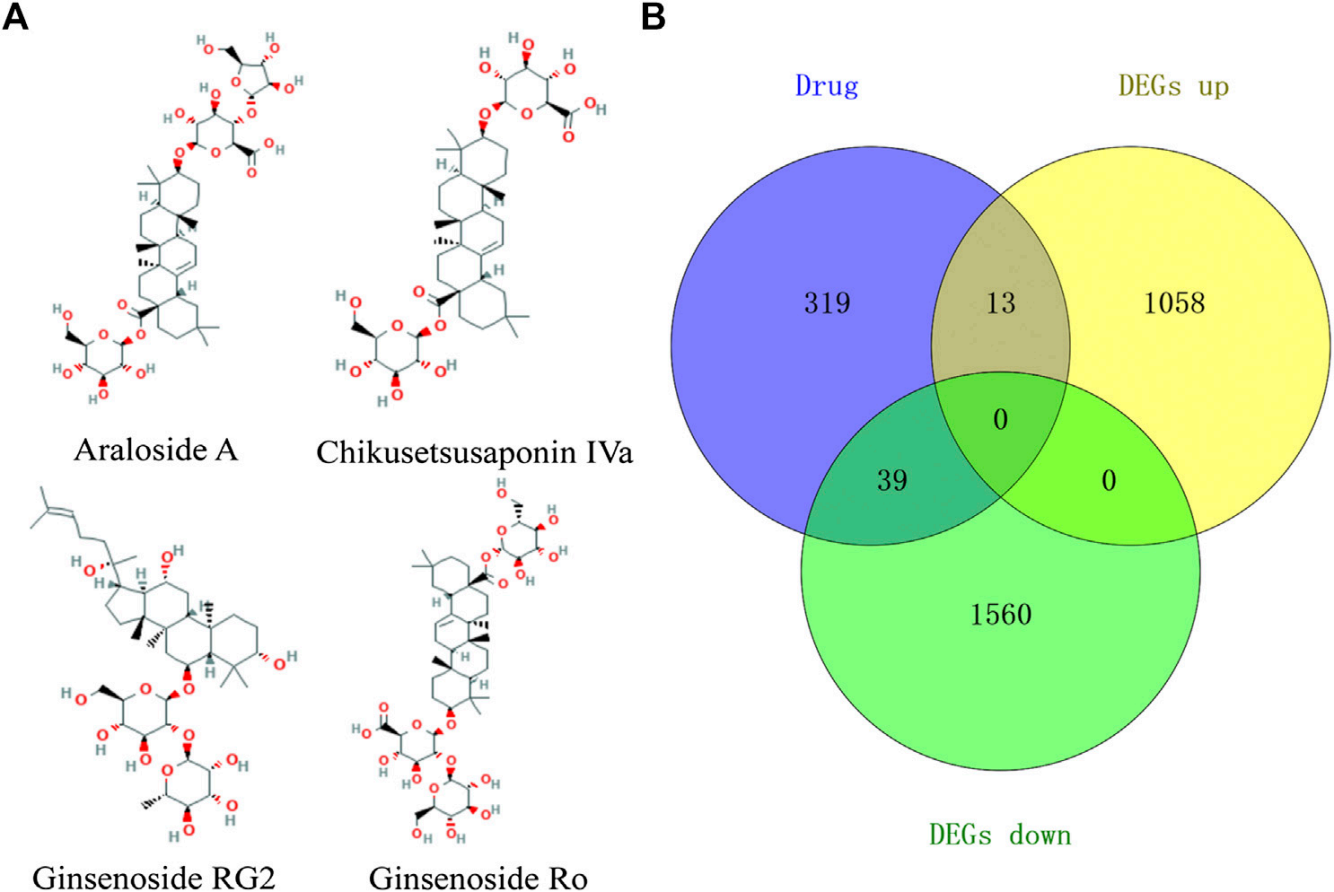
The 2D chemical structure diagram of main ingredients in TSPJ **(A)**, Venn diagram containing the DEGs and drug target gene **(B)**. Note: DEGs up, upregulated genes; DEGs down, downregulated genes.

A total of four active ingredients were identified from TSPJ, including Araloside A, Chikusetsusaponin IVa, Ginsenoside Rg2, and Ginsenoside Ro. The targets of the four main ingredients (Figure 3A) of TSPJ were predicted by using the PharmMapper database and SwissTargetPrediction database. We obtained 157 Araloside A targets, 157 Chikusetsusaponin IVa targets, 163 Ginsenoside Rg2 targets, and 127 Ginsenoside Ro targets. Then, 371 drug targets were attained by using the UniProt database to screen and remove repetition. Subsequently, the drug targets and DEGs were set as a background list to get the overlapping targets using Venn diagrams. As shown in the Venn diagram (Figure 3B), 52 overlapping targets could be tested in further research.

### Protein–Protein Interactions Network

**FIGURE 4:**
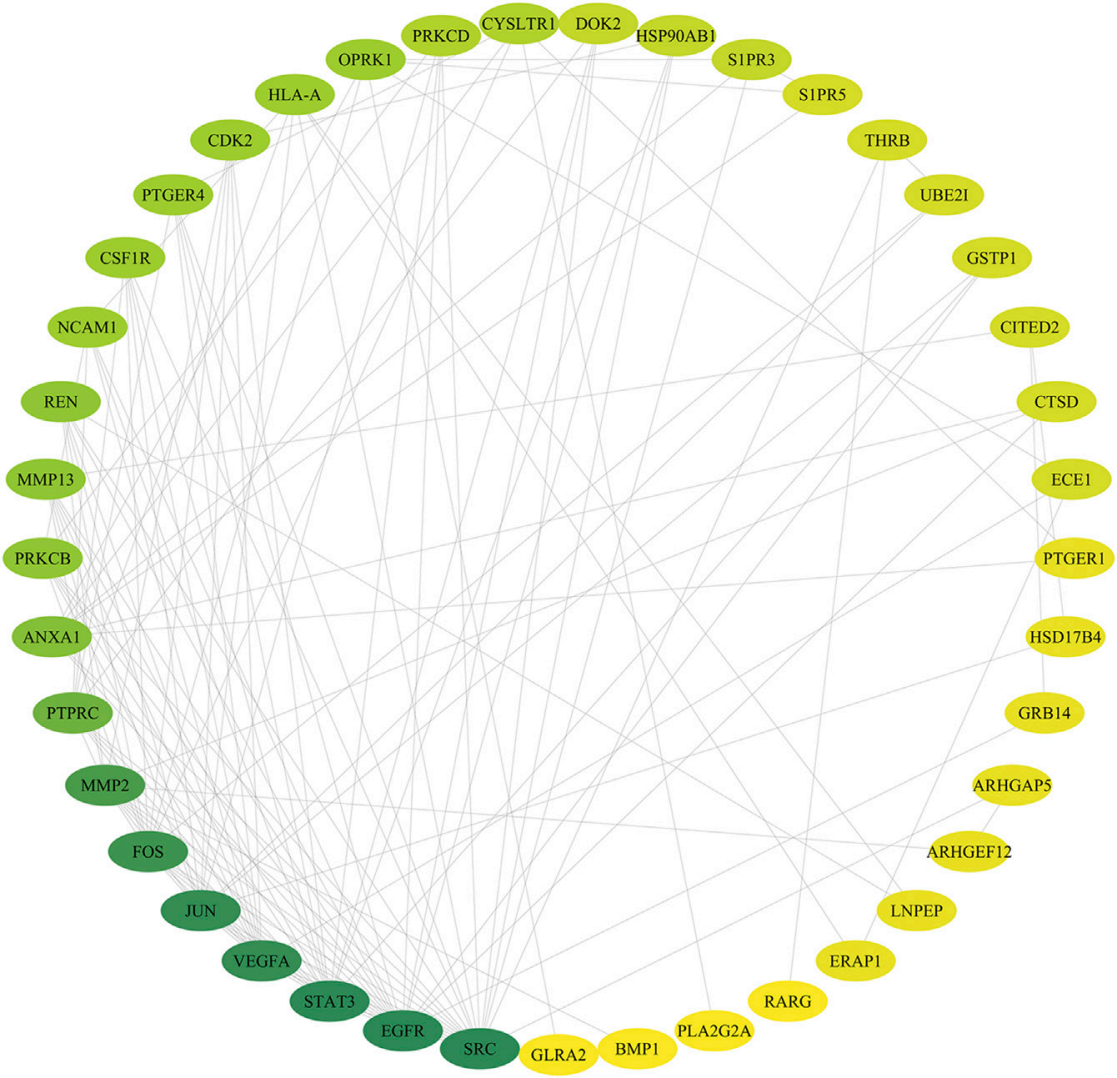
Drug-DEGs PPI network. Note: the color of the nodes is shown in a gradient from green to yellow according to the descending order of the degree value.

According to the related data in the STRING databases, the PPI relationships of the overlapping targets were obtained. Using the Cytoscape3.7.2 software, we constructed the PPI network of the overlapping targets. In the network (Figure 4), nodes represent targets and edges represent interaction relationships existing between two targets. The value of degree indicates the connectivity of the node in the network. The higher the value of the node, the more important the node is in the network. We found a total of 41 nodes and 124 edges in this PPI network after applying the “Network Analyzer” plugin. According to the node degree, some hub targets were identified among the network. The top 10 (degree ≥ 7) targets in the network are SRC proto-oncogene, nonreceptor tyrosine kinase, epidermal growth factor receptor, signal transducer and activator of transcription 3, vascular endothelial growth factor A, jun proto-oncogene, AP-1 transcription factor subunit, Fos proto-oncogene, AP-1 transcription factor subunit, matrix metallopeptidase 2, protein tyrosine phosphatase receptor type C, annexin A1, protein kinase C beta, matrix metallopeptidase 13, and renin.

### Signaling Pathway

**FIGURE 5:**
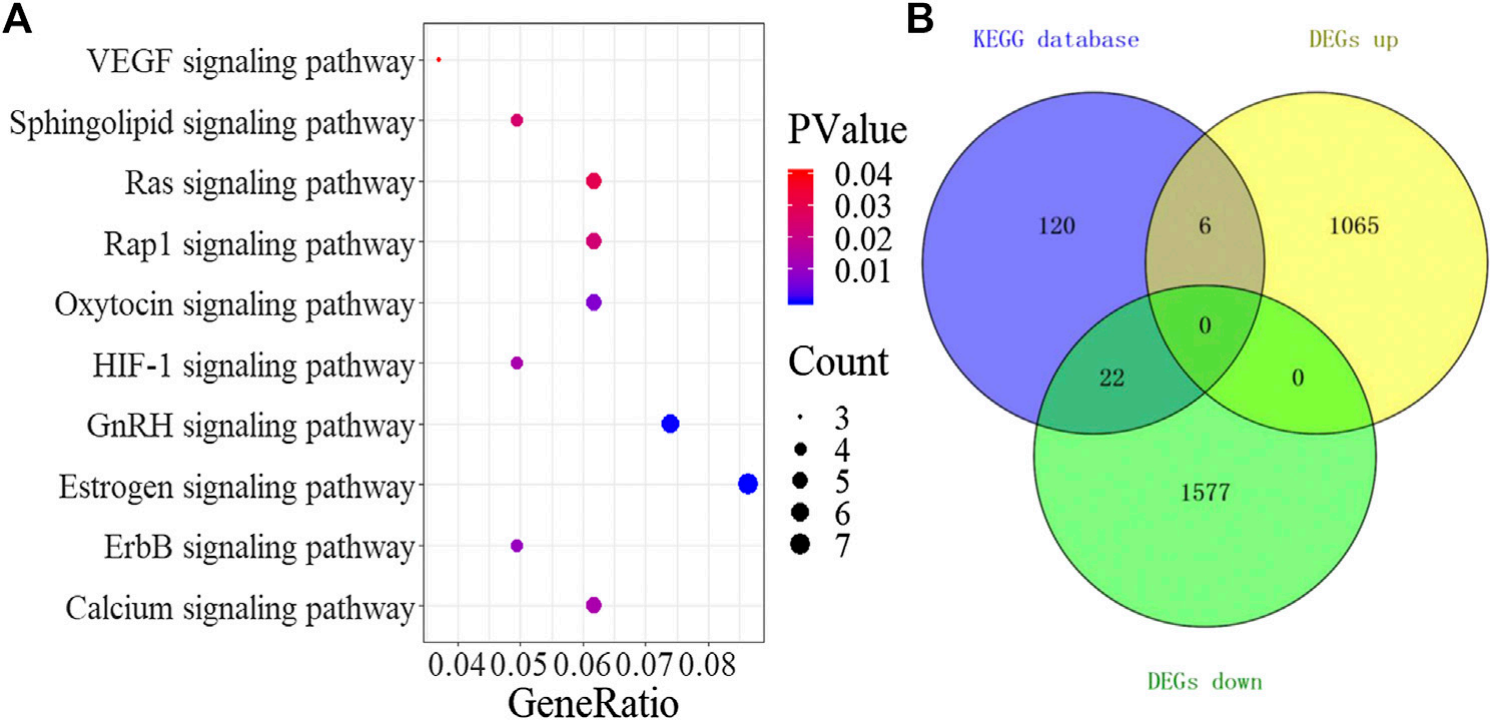
KEGG signaling pathway **(A)**, Venn diagram containing the DEGs and targets in signaling pathways **(B)**.

A total of 10 significant signaling pathways (*p* < 0.05) were obtained by KEGG analysis of the 52 drug and DEGs overlapping targets in the DAVID database. The results of pathway enrichment analysis (Figure 5A) included VEGF, sphingolipid, Ras, Rap1, oxytocin, HIF-1, GnRH, estrogen, ErbB, and calcium signaling pathways. Through the analysis of the pathways, we found that all 10 signaling pathways were related to angiogenesis. Antiangiogenesis may be one of the effective mechanisms of TSPJ in the treatment of RA. Recent studies showed that the VEGF (Elaimy and Mercurio, 2018; Apte et al., 2019) and the HIF-1 signaling pathways (Wang et al., 2016; Serocki et al., 2018) were classic angiogenesis signaling pathways. Next, we downloaded all the genes in the two classic signaling pathways (the VEGF signaling pathway and the HIF-1 signaling pathway) from the KEGG database, and 148 genes were obtained after filtering and deleting the duplicates. Intersection analysis between the 148 genes in the signaling pathways and the DEGs identified 28 genes related to angiogenesis, including six upregulated and 22 downregulated DEGs (Figure 5B). Five out of the top 10 targets (SRC proto-oncogene, nonreceptor tyrosine kinase, epidermal growth factor receptor, signal transducer and activator of transcription 3, vascular endothelial growth factor A, and protein kinase C beta) in the PPI network were also in the same two pathways, and they may play an important role in the mechanism underlying the anti-angiogenesis effect of TSPJ.

### Correlation Analysis

**FIGURE 6:**
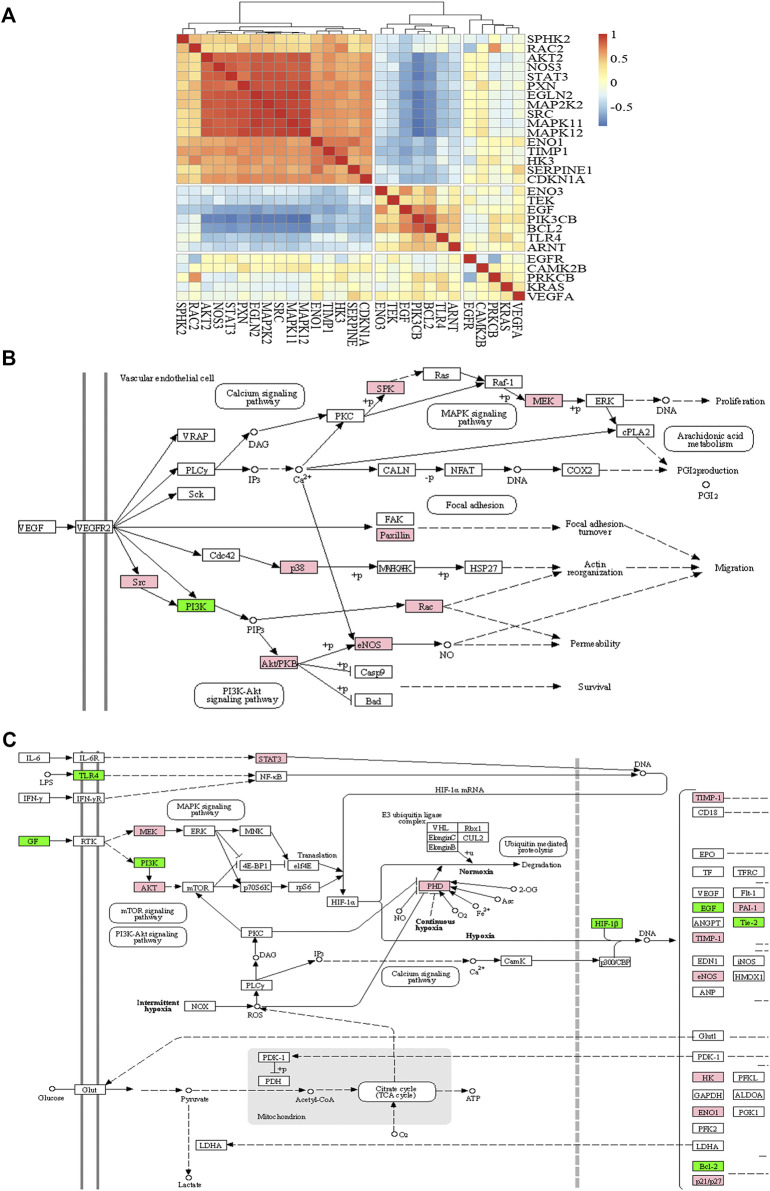
The heat map of correlation analysis **(A)**, VEGF signaling pathway **(B)**, and HIF-1 signaling pathway **(C)**. Note: red or pink represents positive correlation and blue or green represents negative correlation.

To analyze the synergistic mechanisms of the antiangiogenesis effect, we performed a correlation analysis for DEGs in the pathways and calculated the correlation coefficient between the expression of each gene and the others. According to the results of the correlation analysis, the genes with a correlation coefficient greater than 0.5 are defined as positive correlation genes to promote each other, and those with correlation coefficients less than −0.5 are negative correlation genes that inhibit each other. The results of the correlation analysis are shown as a heat map (Figure 6A) by the “Pheatmap” R package. By clustering the results of the correlation analysis, the genes in the red region indicate a positive correlation, the blue region indicates a negative correlation, and the yellow region indicates that the correlation is not significant; seven negative and 16 positive correlation genes were found. We mapped these genes into signaling pathways (Figures 6B,C) by the KEGG database. As shown in the figures, nine genes (sphingosine kinase 2, mitogen-activated protein kinase kinase 2, paxillin, Mitogen-activated protein kinase 11, Mitogen-activated protein kinase 12, SRC proto-oncogene, nonreceptor tyrosine kinase, Ras-related C3 botulinum toxin substrate 2, AKT serine/threonine kinase 2, and nitric oxide synthase 3) showed positive correlations and promoted each other’s expression. PIK3CB is the only suppressed gene in the VEGF signaling pathway. However, in the HIF-1 signaling pathway, seven genes (epidermal growth factor, phosphatidylinositol 4,5-bisphosphate 3-kinase catalytic subunit beta, enolase 3, BCL2 apoptosis regulator, Toll-like receptor 4, aryl hydrocarbon receptor nuclear translocator, and TEK receptor tyrosine kinase) showed negative correlations, and 11 genes (signal transducer and activator of transcription 3, mitogen-activated protein kinase kinase 2, AKT serine/threonine kinase 2, egl-9 family hypoxia inducible factor 2, TIMP metallopeptidase inhibitor 1, serpin family E member 1, nitric oxide synthase 3, hexokinase 3, enolase 1, and cyclin-dependent kinase inhibitor 1A) had positive correlations.

## Discussion

In this study, 52 overlapping genes were found based on 2670 DEGs and 371 drug targets. In addition, the pathways of the KEGG analysis were all involved in angiogenesis, especially the HIF-1α and VEGF signaling pathways. Two key genes in the PPI network were also in these two pathways. Moreover, we successfully established CIA model and verified partly of our computational prediction results. These results suggested that TSPJ could inhibit angiogenesis by targeting the HIF-1α or the VEGF signaling pathway through multitargets such as SRC and STAT 3, effectively treating RA.

Angiogenesis is one of the main features of RA, and it often occurs in the early stage of inflammation (Lu et al., 2017; Li Y. et al., 2018). RA synovial macrophages and fibroblasts produce growth factors, cytokines, and chemokines after inflammatory stimulation or hypoxia (Bartok and Firestein, 2010; Konisti et al., 2012). These include the production of HIF-1α after hypoxia and the most famous angiogenic stimulator VEGF (Świdrowska-Jaros and Smolewska, 2018). New angiogenesis aggravates the inflammation and then promotes synovial neovascularization. There is a positive feedback loop between the two (Leblond et al., 2017). Inhibition of angiogenesis has become a new choice for the treatment of RA in recent years (Jia et al., 2018). However, many drugs targeting RA angiogenesis are still under research and development (Alaarg et al., 2017; Feng and Chen, 2018), including Chinese medicine compounds or monomers. For example, sinomenine inhibits angiogenesis through the HIF-1α-VEGF-ANG-1 axis (Feng et al., 2019). *Tripterygium wilfordii*, Hook f. indirectly suppresses VEGF by inhibiting TNF-α (Zhang W. et al., 2017). It has previously been reported that some components of TSPJ have an antiangiogenic effect in tumors; however, the mechanism of the antiangiogenesis effect of TSPJ in the treatment of RA was not clear. Because of the multicomponent combination therapy, it is difficult to identify the specific interactions between the components and a single target. Network pharmacology is consistent with the overall concept and can help to predict the targets and identify the mechanisms of TCM (Lee et al., 2019; Zhang et al., 2019). We use the methods of network pharmacology and bioinformatics analysis to further understand the possible mechanism underlying the antiangiogenic effect of TSPJ.

In this study, four active components (Araloside A, Chikusetsusaponin IVa, Ginsenoside Rg2, and Ginsenoside Ro) of TSPJ and the 371 potential targets of TSPJ were predicted. DEGs in RA synovium were obtained by difference analysis and could be involved in the pathogenesis of RA. Acting on these DEGs may be very helpful for the targeted therapy of RA. The pathways containing these DEGs may also be important targets for the treatment of RA. The results of the intersection analysis showed that 52 of the targets may be related. The protein interactions of the 52 overlapping genes were obtained from the STRING database and network visualized by Cytoscape3.7.2 software. The top 10 genes' (SRC proto-oncogene, nonreceptor tyrosine kinase, epidermal growth factor receptor, signal transducer and activator of transcription 3, vascular endothelial growth factor A, jun proto-oncogene, AP-1 transcription factor subunit, Fos proto-oncogene, AP-1 transcription factor subunit, matrix metallopeptidase 2, protein tyrosine phosphatase receptor type C, annexin A1, protein kinase C beta, matrix metallopeptidase 13, and renin) degree values were not less than seven according to the network visualization analysis. A high degree value indicates that these genes play a key role in the regulatory network of RA proteins in responding to TSPJ therapy. Similarly, the important pathways of TSPJ in the treatment of RA were found by the KEGG analysis of overlapping genes. To our surprise, the 10 signaling pathways were all related to angiogenesis, and the top four genes (SRC proto-oncogene, nonreceptor tyrosine kinase, epidermal growth factor receptor, signal transducer and activator of transcription 3, and vascular endothelial growth factor A) in the PPI network are all involved in the VEGF and HIF-1 signaling pathways. We supposed that TSPJ might treat RA by inhibiting angiogenesis.

The VEGF and HIF-1 signaling pathways are two important pathways of angiogenesis. VEGF-A induces endothelial cell proliferation, migration, and survival by activating VEGFR2 and its downstream signal transduction pathway, thus promoting new angiogenesis (Karaman et al., 2018). Synovial inflammation and hyperplasia consume much oxygen, leaving the tissue in a state of local hypoxia. When hypoxia occurs, activation of the HIF-1 signal pathway leads to an increase in the expression of angiogenesis-related factors such as VEGF (Li Y. et al., 2018; Fallah and Rini, 2019). To further identify the synergistic mechanism of the TSPJ against angiogenesis, we analyzed the correlation of DEGs in the VEGF and HIF-1 signaling pathways. Seven genes showed a negative correlation, and 16 showed a positive correlation. The difference in correlation reflects the competition or mutual promotion among these genes (Xuan et al., 2019). Meanwhile, we found only two genes (SRC and STAT 3) with higher degree values in angiogenesis-related pathways according to the results of the correlation analysis. STAT 3 is a marker of angiogenesis that interacts with SRC (Sp et al., 2017). Li et al. and L. Claesson-Welsh et al. have shown that the activation of SRC phosphorylation promotes angiogenesis (Claesson-Welsh and Welsh, 2013; Li P. et al., 2018). In addition, the results showed that the degree value of STAT 3 in the PPI network is higher than that of other DEGs in the HIF-1 signaling pathway. Studies have shown that STAT 3 is a potential therapeutic target of RA (Oike et al., 2017). STAT 3 directly regulates VEGF, and blocking STAT 3 can effectively inhibit angiogenesis (Wei et al., 2003; Xu et al., 2005). Therefore, we propose that when SRC and STAT 3 are inhibited by TSPJ, the genes positively related to SRC and STAT 3 are also suppressed, and thus reduce angiogenesis.

To further verify the prediction results above, we successfully established CIA mice model. The data showed that TSPJ could significantly reduce the arthritis index of CIA mice. This indicated that TSPJ can effectively relieve the symptoms of CIA mice. The results of H&E staining and histology score of knee joint sections showed that TSPJ inhibited articular cartilage destruction, synovial hyperplasia, and inflammatory cell infiltration in a dose-dependent manner. In addition, TSPJ could reduce the expression of HIF-1 α and VEGFA in serum. This is consistent with our prediction that TSPJ may act as an antiangiogenic by regulating the VEGF or the HIF-1 signaling pathway. Results also showed that TSPJ could decrease the expression of IL-1β and IL-17A in spleen (Supplementary Figures S2–S5). The above results indicated that TSPJ application might improve joint inflammation, vascular proliferation, and bone erosion.

## Conclusion

The current study demonstrated that TSPJ may regulate the VEGF or HIF-1 signaling pathways by inhibiting two targets, STAT 3 and SRC, thus inhibiting inflammation and angiogenesis, which will provide a new strategy for the treatment of RA.

## Data Availability Statement

All data sets presented in this study are included in the article/Supplementary Material.

## Author Contributions

ZF and ZM conceived and designed the experiments; XG, JJ, XF, and YL analyzed the data; XH provided information support; ZF and XG drafted the manuscript; and ZF, GJ, and ZM amended the final manuscript.

## Funding

This project was supported by grants from the National Natural Science Foundation of China (No. 81703783) and the Hubei Provincial Natural Science Foundation (No. 2017CFB126).

## Conflict of Interest

The authors declare that the research was conducted in the absence of any commercial or financial relationships that could be construed as a potential conflict of interest.

## References

[B1] Alaarg A. Pérez-Medina C. Metselaar J. M. Nahrendorf M. Fayad Z. A. Storm G. (2017). Applying nanomedicine in maladaptive inflammation and angiogenesis. Adv. Drug Deliv. Rev. 119, 143–158. 10.1016/j.addr.2017.05.009 28506745 PMC5682240

[B2] Aletaha D. Smolen J. S. (2018). Diagnosis and management of rheumatoid arthritis. JAMA 320 (13), 1360–1372. 10.1001/jama.2018.13103 30285183

[B3] Apte R. S. Chen D. S. Ferrara N. (2019). VEGF in signaling and disease: beyond discovery and development. Cell 176 (6), 1248–1264. 10.1016/j.cell.2019.01.02 30849371 PMC6410740

[B4] Bartok B. Firestein G. S. (2010). Fibroblast-like synoviocytes: key effector cells in rheumatoid arthritis. Immunol. Rev. 233 (1), 233–255. 10.1111/j.0105-2896.2009.00859.x 20193003 PMC2913689

[B5] Boezio B. Audouze K. Ducrot P. Taboureau O. (2017). Network-based approaches in pharmacology. Molecular informatics 36 (10), 1700048 10.1002/minf.201700048 28692140

[B6] Chen Z. Wang H. Xia Y. Yan F. Lu Y. (2018). Therapeutic potential of mesenchymal cell-derived miRNA-150-5p-expressing exosomes in rheumatoid arthritis mediated by the modulation of MMP14 and VEGF. J. Immunol. 201 (8), 2472–2482. 10.4049/jimmunol.1800304 30224512 PMC6176104

[B7] Choudhary N. Bhatt L. Prabhavalkar K. (2018). Experimental animal models for rheumatoid arthritis. Immunopharmacol. Immunotoxicol. 40 (3), 193–200. 10.1080/08923973.2018.1434793 29433367

[B8] Claesson-Welsh L. Welsh M. (2013). VEGFA and tumour angiogenesis. J. Intern. Med. 273 (2), 114–127. 10.1111/joim.12019 23216836

[B9] Croia C. Bursi R. Sutera D. Petrelli F. Alunno A. Puxeddu I. (2019). One year in review 2019: pathogenesis of rheumatoid arthritis. Clin. Exp. Rheumatol. 37 (3), 347–357. 31111823

[B10] DennisG.Jr.ShermanB. T.HosackD. A.YangJ.GaoW.LaneH. C. (2003). DAVID: database for annotation, visualization, and integrated discovery. Genome Biol. 4 (5), P3 10.1186/gb-2003-4-5-p3 12734009

[B11] Ding Y. Keming L. Changcheng Z. (2008). Study on the anti-inflammatory effect of total saponins of *Panax japonicus* . Hubei J. Tradit. Chin. Med. 4, 7–8. 10.3969/j.issn.1000-0704.2008.04.003

[B12] Ding Y. Qi Y. Changcheng Z. (2009). Effect of total saponins of *Panax japonicus* on expression of serum TNF-α and IL-1β in rheumatoid arthritis rats. Shandong Med. J. 19, 4–6. 10.3969/j.issn.1002-266X.2009.19.002

[B13] Ding Y. Zhao Q. Wang L. (2019). Pro-apoptotic and anti-inflammatory effects of araloside A on human rheumatoid arthritis fibroblast-like synoviocytes. Chem. Biol. Interact. 306, 131–137. 10.1016/j.cbi.2019.04.025 31004595

[B14] Elaimy A. L. Mercurio A. M. (2018). Convergence of VEGF and YAP/TAZ signaling: implications for angiogenesis and cancer biology. Sci. Signal. 11 (552), eaau1165 10.1126/scisignal.aau1165 30327408 PMC6525620

[B15] Elshabrawy H. A. Chen Z. Volin M. V. Ravella S. Virupannavar S. Shahrara S. (2015). The pathogenic role of angiogenesis in rheumatoid arthritis. Angiogenesis 18 (4), 433–448. 10.1007/s10456-015-9477-2 26198292 PMC4879881

[B16] Fallah J. Rini B. I. (2019). HIF inhibitors: status of current clinical development. Curr. Oncol. Rep. 21 (1), 6 10.1007/s11912-019-0752-z 30671662

[B17] Feng X. Chen Y. (2018). Drug delivery targets and systems for targeted treatment of rheumatoid arthritis. J. Drug Target. 26 (10), 845–857. 10.1080/1061186x.2018.1433680 29376442

[B18] Feng Z.-t. Yang T. Hou X.-q. Wu H.-y. Feng J.-t. Ou B.-j. (2019). Sinomenine mitigates collagen-induced arthritis mice by inhibiting angiogenesis. Biomed. Pharmacother. 113, 108759 10.1016/j.biopha.2019.108759 30856539

[B19] Guo Z. Feng Z. Zhang H. Yan L. Liang M. Mei Z. (2019). Research progress of bamboo ginseng and its preparations in the treatment of rheumatoid arthritis. J. Chin. Med. Mater. 42 (4), 941–944. 10.13863/j.issn1001-4454.2019.04.050

[B20] Jia W. Wu W. Yang D. Xiao C. Huang M. Long F. (2018). GATA4 regulates angiogenesis and persistence of inflammation in rheumatoid arthritis. Cell Death Dis. 9 (5), 503 10.1038/s41419-018-0570-5 29717129 PMC5931571

[B21] Karaman S. Leppänen V.-M. Alitalo K. (2018). Vascular endothelial growth factor signaling in development and disease. Development 145 (14), dev151019 10.1242/dev.151019 30030240

[B22] Konisti S. Kiriakidis S. Paleolog E. M. (2012). Hypoxia--a key regulator of angiogenesis and inflammation in rheumatoid arthritis. Nat. Rev. Rheumatol. 8 (3), 153–162. 10.1038/nrrheum.2011.205 22293762

[B23] Leblond A. Allanore Y. Avouac J. (2017). Targeting synovial neoangiogenesis in rheumatoid arthritis. Autoimmun. Rev. 16 (6), 594–601. 10.1016/j.autrev.2017.04.005 28414154

[B24] Lee W.-Y. Lee C.-Y. Kim Y.-S. Kim C.-E. (2019). The methodological trends of traditional herbal medicine employing network pharmacology. Biomolecules 9 (8), 362 10.3390/biom9080362 PMC6723118 31412658

[B25] Li P. Chen D. Cui Y. Zhang W. Weng J. Yu L. (2018). Src plays an important role in AGE-induced endothelial cell proliferation, migration, and tubulogenesis. Front. Physiol. 9, 765 10.3389/fphys.2018.00765 29977209 PMC6021521

[B26] Li Y. Liu Y. Wang C. Xia W.-R. Zheng J.-Y. Yang J. (2018). Succinate induces synovial angiogenesis in rheumatoid arthritis through metabolic remodeling and HIF-1α/VEGF axis. Free Radic. Biol. Med. 126, 1–14. 10.1016/j.freeradbiomed.2018.07.009 30030103

[B27] Lu Y. Yu S. S. Zong M. Fan S. S. Lu T. B. Gong R. H. (2017). Glucose-6-Phosphate isomerase (G6PI) mediates hypoxia-induced angiogenesis in rheumatoid arthritis. Sci. Rep. 7, 40274 10.1038/srep40274 28067317 PMC5220294

[B28] Oike T. Sato Y. Kobayashi T. Miyamoto K. Nakamura S. Kaneko Y. (2017). Stat3 as a potential therapeutic target for rheumatoid arthritis. Sci. Rep. 7 (1), 10965 10.1038/s41598-017-11233-w 28887478 PMC5591217

[B29] Ramharack P. Soliman M. E. S. (2018). Bioinformatics-based tools in drug discovery: the cartography from single gene to integrative biological networks. Drug Discov. Today 23 (9), 1658–1665. 10.1016/j.drudis.2018.05.041 29864527

[B30] Sayers E. W. Beck J. Brister J. R. Bolton E. E. Canese K. Comeau D. C. (2020). Database resources of the National Center for Biotechnology Information. Nucleic Acids Res. 48 (D1), D9–D16. 10.1093/nar/gkz899 31602479 PMC6943063

[B31] Scott D. L. Wolfe F. Huizinga T. W. (2010). Rheumatoid arthritis. Lancet 376 (9746), 1094–1108. 10.1016/s0140-6736(10)60826-4 20870100

[B32] Serocki M. Bartoszewska S. Janaszak-Jasiecka A. Ochocka R. J. Collawn J. F. Bartoszewski R. (2018). miRNAs regulate the HIF switch during hypoxia: a novel therapeutic target. Angiogenesis 21 (2), 183–202. 10.1007/s10456-018-9600-2 29383635 PMC5878208

[B33] Sidders B. Karlsson A. Kitching L. Torella R. Karila P. Phelan A. (2018). Network-based drug discovery: coupling network pharmacology with phenotypic screening for neuronal excitability. J. Mol. Biol. 430 (18 Pt A), 3005–3015. 10.1016/j.jmb.2018.07.016 30030026

[B34] Smolen J. S. Aletaha D. Mclnnes I. B. (2016). Rheumatoid arthritis. Lancet 388 (10055), 2023–2038. 10.1016/s0140-6736(16)30173-8 27156434

[B35] Sp N. Kang D. Joung Y. Park J. Kim W. Lee H. (2017). Nobiletin inhibits angiogenesis by regulating src/FAK/STAT3-mediated signaling through PXN in ER⁺ breast cancer cells. Int. J. Mol. Sci. 18 (5), 935 10.3390/ijms18050935 PMC5454848 28468300

[B36] Świdrowska-Jaros J. Smolewska E. (2018). A fresh look at angiogenesis in juvenile idiopathic arthritis. Cent. Eur. J. Immunol. 43 (3), 325–330. 10.5114/ceji.2018.80052 30863199 PMC6410962

[B37] The UniProt Consortium (2017). UniProt: the universal protein knowledgebase. Nucleic Acids Res. 45 (D1), D158–D169. 10.1093/nar/gkw1099 27899622 PMC5210571

[B38] Wang J. Wong Y. K. Liao F. (2018). What has traditional Chinese medicine delivered for modern medicine? Expert. Rev. Mol. Med. 20, e4 10.1017/erm.2018.3 29747718

[B39] Wang L.-H. Jiang X.-R. Yang J.-Y. Bao X.-F. Chen J.-L. Liu X. (2016). SYP-5, a novel HIF-1 inhibitor, suppresses tumor cells invasion and angiogenesis. Eur. J. Pharmacol. 791, 560–568. 10.1016/j.ejphar.2016.09.027 27664769

[B40] Wei D., Le X., Zheng L. Wang L. Frey J. A. Gao A. C., (2003). Stat3 activation regulates the expression of vascular endothelial growth factor and human pancreatic cancer angiogenesis and metastasis. Oncogene 22 (3), 319–329. 10.1038/sj.onc.1206122 12545153

[B41] Xu Q. Briggs J. Park S. Niu G. Kortylewski M. Zhang S. (2005). Targeting Stat3 blocks both HIF-1 and VEGF expression induced by multiple oncogenic growth signaling pathways. Oncogene 24 (36), 5552–5560. 10.1038/sj.onc.1208719 16007214

[B42] Xuan C. Gao Y. Jin M. Xu S. Wang L. Wang Y. (2019). Bioinformatic analysis of Cacybp-associated proteins using human glioma databases. IUBMB Life 71 (7), 827–834. 10.1002/iub.1999 30762928

[B43] Yang B. R. Yuen S. C. Fan G. Y. Cong W. H. Leung S. W. Lee S. M. (2018). Identification of certain Panax species to be potential substitutes for Panax notoginseng in hemostatic treatments. Pharmacol. Res. 134, 1–15. 10.1016/j.phrs.2018.05.005 29772270

[B44] Yang X. Wang R. Zhang S. Zhu W. Tang J. Liu J. (2014). Polysaccharides from *Panax japonicus* C.A. Meyer and their antioxidant activities. Carbohydr. Polym. 101, 386–391. 10.1016/j.carbpol.2013.09.038 24299787

[B45] Yougui L. (2011). The mechanistic approach of Saponins from *Panax japonicus* for treatment of alcohol-induced hepatic iniury *.* Dissertation Hangzhou, China: Zhejiang University.

[B46] Yuan H. Ma Q. Cui H. Liu G. Zhao X. Li W. (2017). How can synergism of traditional medicines benefit from network pharmacology? Molecules 22 (7), 1135 10.3390/molecules22071135 PMC6152294 28686181

[B47] Yu-min H. Ke A. Chun-xi H. Chang-cheng Z. Ding Y. San-jin C. (2019). Triterpene saponins in *Panax japonicus* and their 13C-NMR spectroscopic characteristics. China J. Chin. Mater. Med. 44 (02), 249–260. 10.19540/j.cnki.cjcmm.20181101.016 30989941

[B48] Zhang R.-z. Yu S.-j. Bai H. Ning K. (2017). TCM-Mesh: the database and analytical system for network pharmacology analysis for TCM preparations. Sci. Rep. 7 (1), 2821 10.1038/s41598-017-03039-7 28588237 PMC5460194

[B49] Zhang W. Li F. Gao W. (2017). *Tripterygium wilfordii* inhibiting angiogenesis for rheumatoid arthritis treatment. J. Natl. Med. Assoc. 109 (2), 142–148. 10.1016/j.jnma.2017.02.007 28599756

[B50] Zhang R. Zhu X. Bai H. Ning K. (2019). Network pharmacology databases for traditional Chinese medicine: review and assessment. Front. Pharmacol. 10, 123 10.3389/fphar.2019.00123 30846939 PMC6393382

[B51] Zheng S.-W. Xiao S.-Y. Wang J. Hou W. Wang Y.-P. (2019). Inhibitory effects of Ginsenoside Ro on the growth of B16F10 melanoma via its metabolites. Molecules 24 (16), 2985 10.3390/molecules24162985 PMC6721120 31426477

