# Supplementary material for: Computational Prediction of Antiangiogenesis Synergistic Mechanisms of Total Saponins of Panax japonicus Against Rheumatoid Arthritis
Source: Front Pharmacol. 2020 Oct 29;11:566129. doi: 10.3389/fphar.2020.566129 (PMC7723436; doi:10.3389/fphar.2020.566129)
Supplement: Supplementary file 1 [file Table1_v1.DOCX]

***Supplementary Material***

***1.Determination of active components in TSPJ***

Panax japonicus (T.Nees) C.A.Mey was purchased from the planting base of Wu Feng country (Yichang, China). In the previous experiment, our research group analyzed the active saponins components in Panax japonicus (T.Nees) C.A.Mey by Agilent 1260 Infinity high performance liquid chromatography (HPLC) system (Santa Clara, USA). The column temperature was set at 30°C. The mobile phase consisted of 0.1% phosphoric acid and acetonitrile, was used for isocratic elution at a flow rate of 1.0 ml/min. A total of 5 μl Panax japonicus (T.Nees) C.A.Mey sample extract was injected into Welch Ultimate XB-C18 column (4.6 mm×150 mm, 5μm) and the eluent was monitored at 200 nm for the UV absorption. The peaks on the HPLC fingerprint were compared with standards, and four ginsenosides (Araloside A, Chikusetsusaponin IVa, Ginsenoside Rg2, and Ginsenoside Ro) were identified (**Supplementary Figure 2C and Table 1**).

**
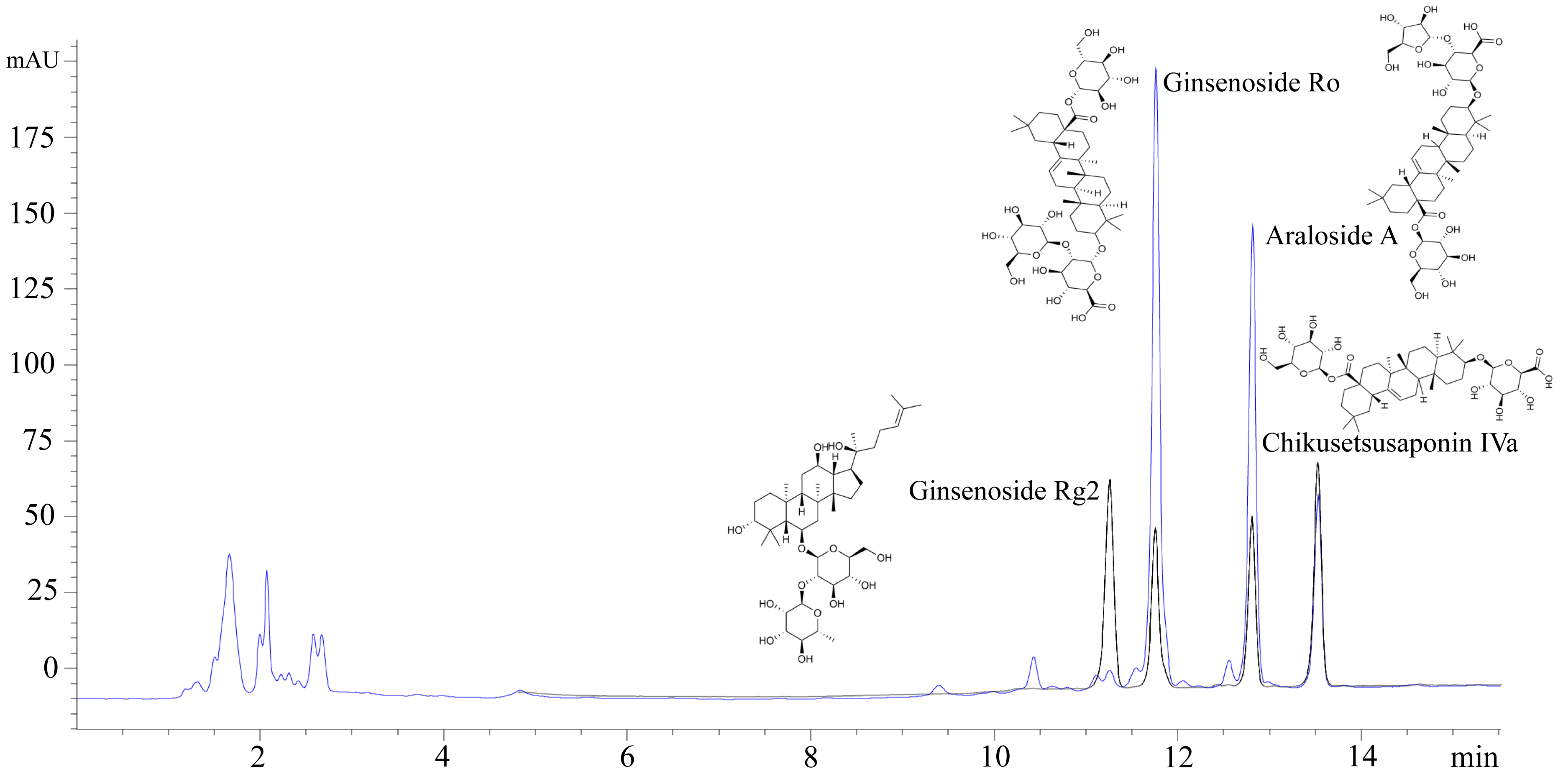
Supplementary Figure 1.** HPLC fingerprint of Panax japonicus (T.Nees) C.A.Mey (1mg/ml, blue) and mixture of four kinds of saponins(100 μg/mL, black).

**Supplementary Table 1.** Information of saponins components in HPLC analysis

| Identified components | Molecular | Contents (μg/mg) |
| --- | --- | --- |
| Ginsenoside Rg2 | C_42_H_72_O_13_ | 3.28018 |
| Ginsenoside Ro | C_48_H_76_O_19_ | 341.62147 |
| Araloside A | C_47_H_74_O_18_ | 244.46698 |
| Chikusetsusaponin IVa | C_42_H_66_O_14_ | 75.52671 |

***2. Data sets***

All samples were taken from the synovium of the joints.

**Supplementary Table 2.** Details of microarray datasets used in this study

| Datasets | GPL | HC | RA |
| --- | --- | --- | --- |
| GSE48780 | GPL570 | 0 | 83 |
| GSE77298 | GPL570 | 7 | 16 |

***3. Methods***

***3.1 Experimental design, CIA model***

All animal studies were performed with the protocol approved by the Ethical Committee for Animal Experiments of China Three Gorges University. DBA/1J male mice were purchased from Changzhou Cavens Lab Animal Co., Ltd (Changzhou, China).

20 DBA/1J mice (male, 20± 1g) were randomly divided into control group (n=5), CIA group (n=5), CIA-TSPJ 50mg/kg group (n=5), and CIA-TSPJ 150mg/kg group (n=5). All the mice from these groups received additional treatments between day 27 and day 37. The control group and CIA group were treated with saline. The treatment group was gavaged with different doses of TSPJ (50mg/kg or 150mg/kg). The mice were anesthetized and then sacrificed on day 38. A total of 15 mice were induced as CIA mice model by a well-established method (Brand et al., 2007).

***3.2 Arthritis assessment***

Joint swelling was considered to be a sign of successful CIA. The arthritis index was scored and recorded every 2 days. Each claw evaluates the arthritis index by visual score, and the evaluation graded as follows: 0) normal; 1) slight swelling and erythema on the tarsals or ankle joint; 2) erythema and mild swelling extending from the ankle to the tarsals; 3) erythema and moderate swelling extending from ankle to metatarsal joints; 4) erythema and severe swelling encompass the ankle, foot, and digits, or ankylosis of the limb (Jia et al., 2019). Each paw was graded and the arthritis index was the sum of the scores for the four paws. The maximal arthritis index of per mouse was 16.

***3.3 Histopathological assessment***

Mice were sacrificed on day 38. The knee joints were removed from mouse corpses and fixed in 4% paraformaldehyde. Then joints were decalcified in ethylenediamine tetraacetic acid (EDTA) for 28 days. Thereafter, the samples were embedded in paraffin and to be sectioned as 4μm sections. Each section was stained with hematoxylin and eosin (H&E). The images of joint sections were obtained with an OLYMPUS microscope. The scores of section were completed by 2 independent observers under the condition of blind. The sections were measured using a scale of 0–4 for grading the synovial inflammation, cartilage damage, and bone erosion (Mukai et al., 2015).

***3.4*** ***Enzyme linked immunosorbent assay***

The mice were sacrificed and collected peripheral blood samples on day 38. The sample was placed in a centrifuge tube and the serum was obtained by centrifugation 20 min at 2000 ~ 3000rpm. All ELISA kits were purchased from the Elabscience Biotechnology Company (Wuhan, China) and detection process according to the manufacturer’s instructions.

***3.5 Real-time quantitativ PCR***

Spleens were homogenized in TRIzol reagent and RNA was extracted according to the manufacturer’s protocol. The cDNA synthesis kit was used to reverse transcribed the RNA of each sample. The primers were added to the instrument for real-time polymerase chain reaction. After 40 cycles, the gene expression was detected and calculated. The primer sequences (forward and reverse) were as follows: for IL-1β, forward: 5’-TGG ACC TTC CAG GA T GAG GAC A-3’ and reverse: 5’-GTT CA T CTC GGA GCC TGT AGT G-3’; for IL-17, forward: 5’-AGC TGG ACC ACC ACA TGA A-3’ and reverse: 5’-AAA CGT GGG GGT TTC TTA GG-3’; for Actin, forward: 5’-AGA GGG AAA TCG TGC GTG AC-3’ and reverse: 5’-CAA TAG TGA TGA CCT GGC CGT-3’.

***3.6 Statistical analysis***

Significant differences were assessed using IBM SPSS Statistics 22.0 software and all data were reported as mean ± standard deviation (SD). One-way ANOVA followed by Dunnett’s t3 test and least significant difference (LSD) test was used to compare the differences of means among the groups. *P* values below 0.05 were considered statistically significant.

***4. Results***

***4.1 TSPJ attenuated the symptoms of CIA mice***

The results showed that TSPJ could relieve the symptoms of CIA mice (**Supplementary Figure 2)**. Compared with the control group, the arthritis index of the CIA group was significantly increased (*P* < 0.01). Compared with the CIA group, there was statistical difference in the CIA-TSPJ 150 mg/kg group on day 32 (*P* < 0.05) and the CIA-TSPJ 50 mg/kg group on day 34 (*P* < 0.01) (**Supplementary Figure 2)**.


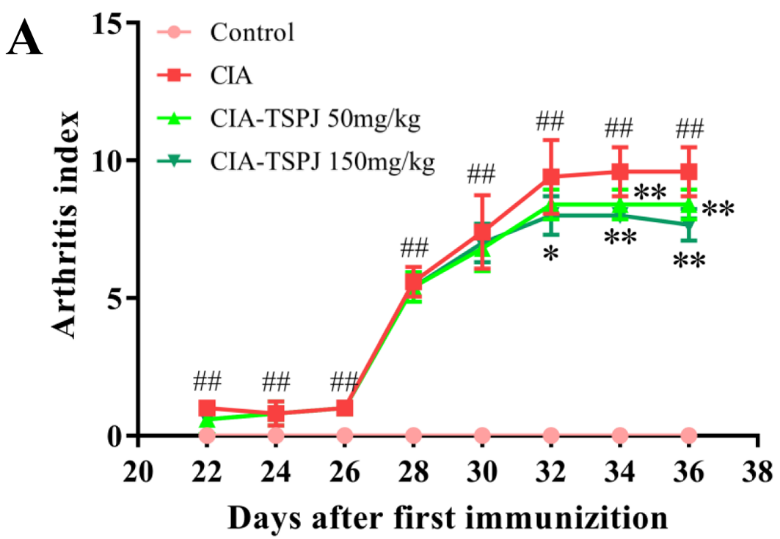


**Supplementary Figure 2.** TSPJ attenuated the symptoms of CIA mice. ^#^*P* < 0.05 &^##^*P* < 0.01 vs Control group, **P* < 0.05&***P* < 0.01 vs CIA group.

***4.2 TSPJ protects knee joint of CIA mice***


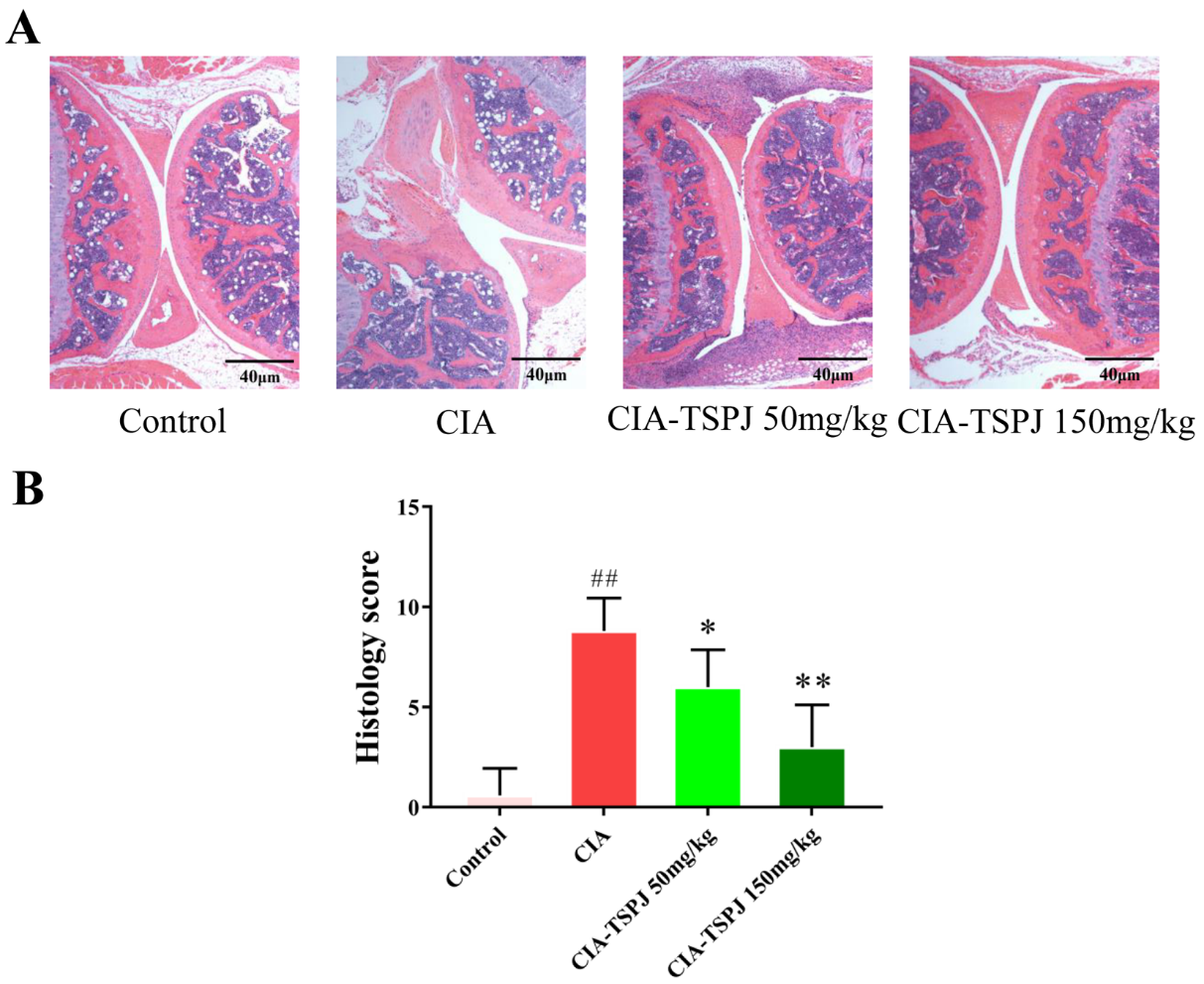
The results showed that the articular cavity of the CIA group was infiltrated with a large number of inflammatory cells and badly damaged, which was compared with the control group (**Supplementary Figure 3A**). The symptoms of mice treated with TSPJ were significantly improved. The results of the histological assessment showed that TPSJ can reduce histological score (*P* < 0.05) in a dose-dependent manner (**Supplementary Figure 3B**).

**Supplementary Figure 3.** TSPJ protects knee joint of CIA mice. The knee joints stained with H&E (A) The histopathological severity were assessed and calculated (B) Data are shown as mean ± SD of three independent experiments. Scale bar=40μm. ^#^*P* < 0.05 &^##^*P* < 0.01 vs Control group, **P* < 0.05&***P* < 0.01 vs CIA group.

***4.3 ELISA detected the expression of related factors***

The expression levels of VEGF-A and HIF-1α in the CIA group were significantly higher than those in the control group (*P* < 0.01). However, different doses of TSPJ can effectively reduce the expression levels of VEGF-A and HIF-α (*P* < 0.01) (**Supplementary Figure 4**).


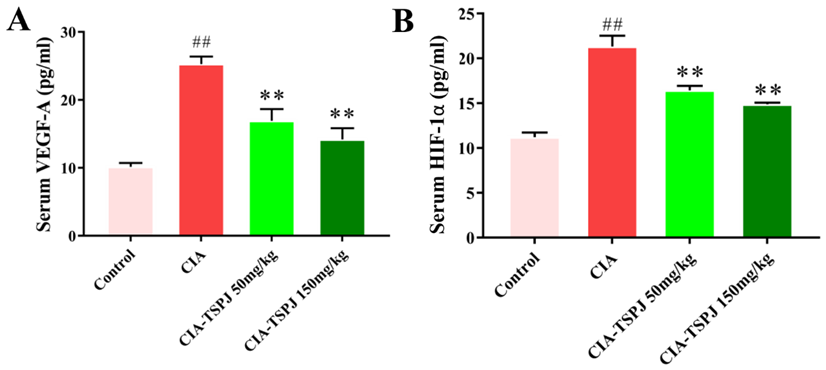


**Supplementary Figure 4.** The serum level of VEGF-A (A) and HIF-1α (B) were determined using the ELISA. Data are shown as mean ± SD of three independent experiments. ^#^*P* < 0.05 &^##^*P* < 0.01 vs Control group, **P* < 0.05&***P* < 0.01 vs CIA group.

***4.4*** ***mRNA expression levels of IL-1β and IL-17A***

The results showed that the levels of IL-1β and IL-17A in the CIA group were significantly higher than those in the control group (*P* < 0.01). The expression levels of IL-1βand IL-17A in TSPJ treated groups were significantly lower than those of the CIA group (*P* < 0.01), and the effect of high dose was better than that of low dose (**Supplementary Figure 5**).

***
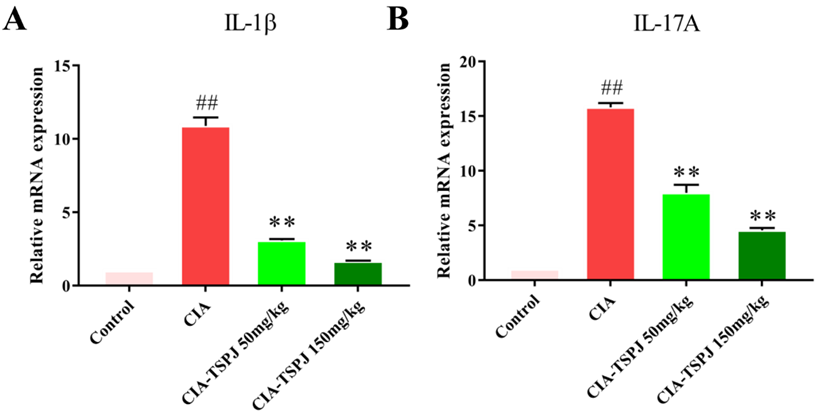
***

**Supplementary Figure 5.** The levels of IL-1β (A) and IL17A (B) were detected by qRT-PCR. Data are shown as mean ± SD of three independent experiments. ^#^*P* < 0.05 &^##^*P* < 0.01 vs Control group, **P* < 0.05&***P* < 0.01 vs CIA group.

***References***

Brand, D.D., Latham, K.A., and Rosloniec, E.F. (2007). Collagen-induced arthritis. *Nat Protoc* 2(5)**,** 1269-1275. doi: 10.1038/nprot.2007.173.

Jia, Q., Wang, T., Wang, X., Xu, H., Liu, Y., Wang, Y., et al. (2019). Astragalin Suppresses Inflammatory Responses and Bone Destruction in Mice With Collagen-Induced Arthritis and in Human Fibroblast-Like Synoviocytes. *Front Pharmacol* 10**,** 94. doi: 10.3389/fphar.2019.00094.

Mukai, T., Gallant, R., Ishida, S., Kittaka, M., Yoshitaka, T., Fox, D.A., et al. (2015). Loss of SH3 domain-binding protein 2 function suppresses bone destruction in tumor necrosis factor-driven and collagen-induced arthritis in mice. *Arthritis Rheumatol* 67(3)**,** 656-667. doi: 10.1002/art.38975.
